# A novel potential inflammation-nutrition biomarker for predicting lymph node metastasis in clinically node-negative colon cancer

**DOI:** 10.3389/fonc.2023.995637

**Published:** 2023-04-04

**Authors:** Wanyao Duan, Wei Wang, Chiyi He

**Affiliations:** Department of Gastroenterology, Yijishan Hospital of Wannan Medical College, Wuhu, Anhui, China

**Keywords:** colon cancer, lymph node metastasis, (platelet × albumin)/lymphocyte ratio, restricted cubic spline, sensitivity analysis

## Abstract

**Background:**

The purpose of this study is to investigate the predictive significance of (platelet × albumin)/lymphocyte ratio (PALR) for lymph node metastasis (LNM) in patients with clinically node-negative colon cancer (cN0 CC).

**Methods:**

Data from 800 patients with primary CC who underwent radical surgery between March 2016 and June 2021 were reviewed. The non-linear relationship between PALR and the risk of LNM was explored using a restricted cubic spline (RCS) function while a receiver operating characteristic (ROC) curve was developed to determine the predictive value of PALR. Patients were categorized into high- and low-PALR cohorts according to the optimum cut-off values derived from Youden’s index. Univariate and multivariate logistic regression analyses were used to identify the independent indicators of LNM. Sensitivity analysis was performed to repeat the main analyses with the quartile of PALR.

**Results:**

A total of eligible 269 patients with primary cN0 CC were retrospectively selected. The value of the area under the ROC curve for PALR for predicting LNM was 0.607. RCS visualized the uptrend linear relationship between PALR and the risk of LNM (p-value for non-linearity > 0.05). PALR (odds ratio = 2.118, 95% confidence interval, 1.182-3.786, p = 0.011) was identified as an independent predictor of LNM in patients with cN0 CC. A nomogram incorporating PALR and other independent predictors was constructed with an internally validated concordance index of 0.637. The results of calibration plots and decision curve analysis supported a good performance ability and the sensitivity analysis further confirmed the robustness of our findings.

**Conclusion:**

PALR has promising clinical applications for predicting LNM in patients with cN0 CC.

## Introduction

Colon cancer (CC) is a common malignancy of the gastrointestinal tract and the third leading cause of cancer-related mortality worldwide (1). A large number of studies have shown that lymph node metastasis (LNM) is an independent risk factor for the prognosis of patients with CC (2, 3). In addition, the scope of surgery for patients with CC should take full account of the preoperative lymph node status to avoid overtreatment (4). Furthermore, neoadjuvant chemotherapy is recommended for patients with stage cT1-4N+M0 CC, which can promote tumor regression and improve survival in patients with CC after borderline negative resection (5–8). Therefore, accurate preoperative prediction of LNM is essential for individualized treatment decisions and prognostic assessment (9). Although widely used in clinical practice, imaging techniques have not produced satisfactory results in the preoperative assessment of lymph node status in CC. It has been estimated that about 30% of lymph node involvement is missed by preoperative abdominal contrast-enhanced computed tomography (CECT) (10, 11). The clinical application of several new molecular biomarkers discovered for detecting LNM in CC, such as FXYD3 and miR-323a-3p, seems unrealistic due to their high cost and technical complexity (12, 13). Therefore, there is an urgent need for cost-effective and convenient preoperative biomarkers to accurately assess the LNM in CC. It has been clarified that preoperative inflammatory biomarkers, including platelet/lymphocyte ratio (PLR), neutrophil/lymphocyte ratio (NLR), and albumin (ALB) can predict LNM in patients with CC (14–16). To our knowledge, the clinical significance of (platelet × albumin)/lymphocyte ratio (PALR), has not been assessed in cancers. The purpose of this study was to investigate the predictive value of PALR for LNM in patients with clinically nodal-negative CC (cN0 CC) and develop a nomogram to assist clinicians in formulating individualized treatments.

## Materials and methods

### Patients

**Figure 1:**
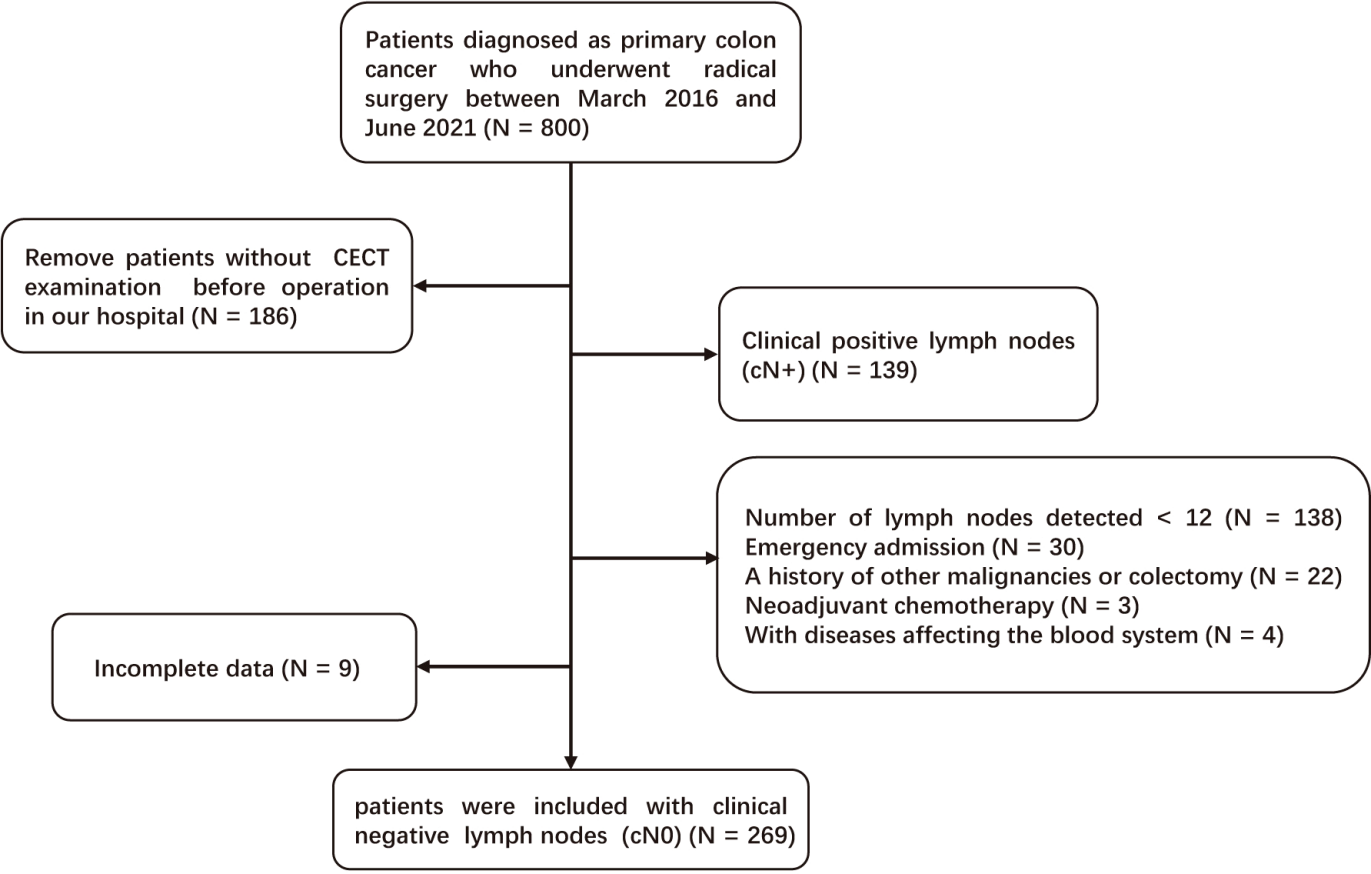
The screening flowchart.

A total of eligible 269 patients with primary cN0 CC who underwent radical surgery between March 2016 and June 2021 in Yijishan Hospital were retrospectively selected. The inclusion criteria were as follows: [1] CC was confirmed by pathological examination; [2] patients underwent curative surgery (R0) and lymph node dissection; and [3] CECT was performed before operation in our hospital. Patients who met the following criteria were excluded: [1] LNM was detected by CECT before operation (cN+); [2] insufficient number of detected lymph nodes (< 12); [3] emergency admission; [4] a history of other malignancies or colectomy; [5] neoadjuvant chemotherapy; [6] with diseases affecting the blood system; and [7] incomplete data. **Figure 1** demonstrates the detailed screening process.

### Data collection

We recorded the serum levels of lymphocytes, platelets, ALB, and carcinoembryonic antigen (CEA) by performing routine blood tests on the day of admission. PLR and PALR were calculated according to the following formulas: PLR = Platelets (10^9^/L)/Lymphocytes (10^9^/L), PALR = (Platelets (10^9^/L) × ALB (g/L))/(Lymphocytes (10^9^/L)/1000). Postoperative pathology results including tumor site, size, grade, and the depth of tumor invasion (T stage) were reviewed by senior pathologists in our hospital. All patients were staged according to the 8^th^ edition of the American Joint Committee on Cancer (AJCC) staging system (17).

### Statistical analysis

Continuous variables were expressed as medians (interquartile range) and analyzed by Mann-Whitney U tests, while categorical variables were expressed as numbers and analyzed by the Chi-square test (18). Comparisons of the values of area under the receiver operating characteristic (ROC) curve (AUC) were carried out using the DeLong test (19, 20). Youden’s index was used to identify the optimum cut-off values of PALR and PLR based on the ROC curves (21). Independent predictors of LNM were obtained by combining univariate and multivariate logistic regression analyses. We used a restricted cubic spline (RCS) function with three knots at the 5^th^, 50^th^, and 95^th^ centiles to develop a flexible model of the association of PALR with the risk of LNM (22). In terms of sensitivity analysis, we incorporated the quartile of PALR in the multivariate analysis. We used AUC, decision curve analysis (DCA), and the calibration curves to evaluate the nomogram as previously described (23–25). The internal validation of the model was performed *via* a bootstrap resample approach (1000 samples), together with the calculation of a corrected concordance index (C-index) (26). SPSS (Version 26.0), MedCalc (Version 15.2), and R (Version 4.0.2) were used for statistical analyses and graphics. All two-sided p values < 0.05 were considered significant.

## Results

### Patients’ baseline characteristics

**Figure 2:**
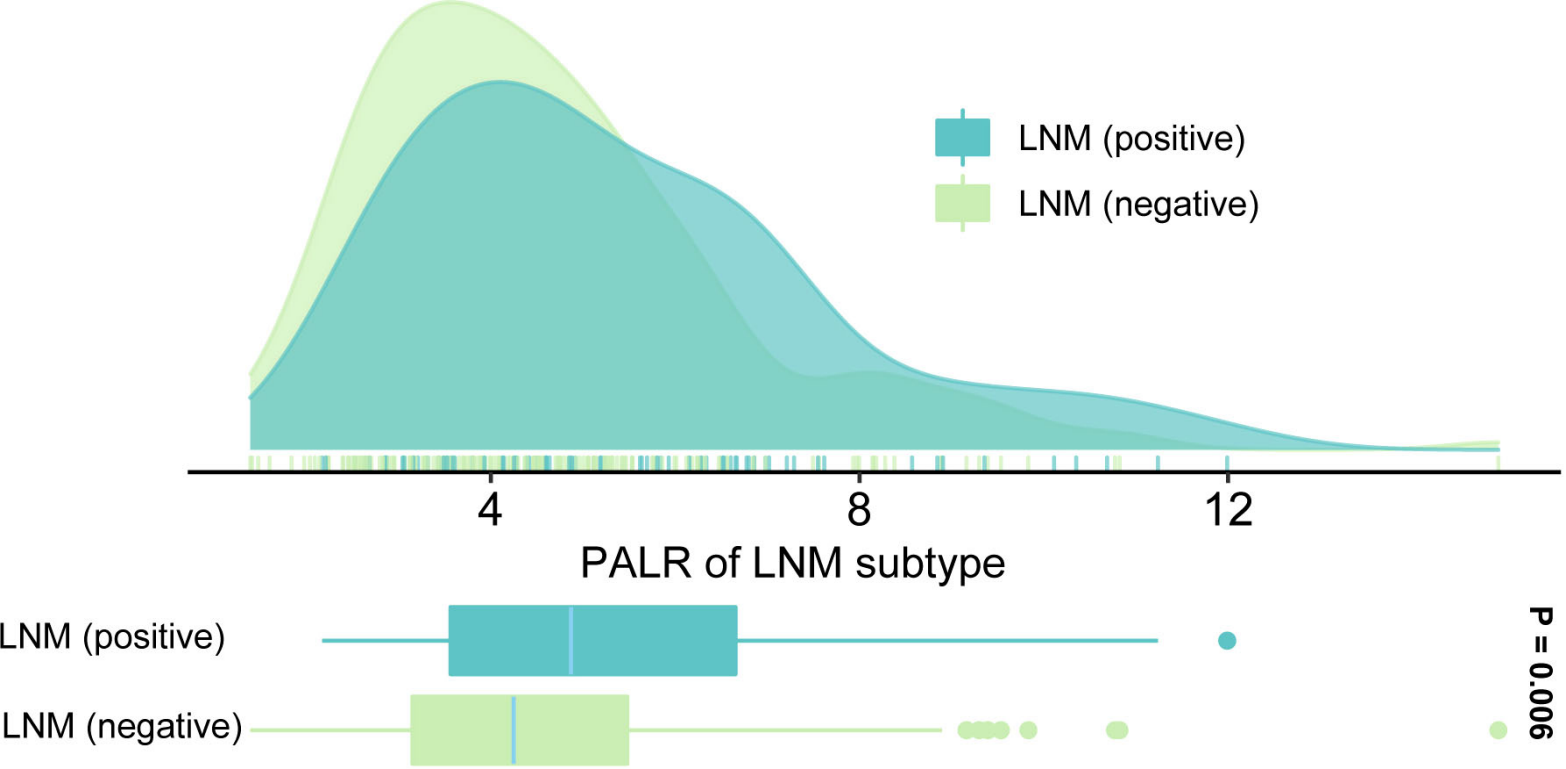
Distribution of PALR values in LNM-positive and LNM-negative groups.

We enrolled 269 eligible patients in our study with a 28.6% LNM rate. The optimum cut-off values of the PALR and PLR for LNM were set to 5.62, and 80.95, respectively. The patients were divided into high- and low- groups according to the optimum cut-off values. Young patients accounted for 52.0% and more than half of the patients were male (58.7%). The location of the tumor was mostly on the left side of the colon (61.7%). The majority of the patients were in T3 and T4 stages (81.0%) and almost all patients had highly or moderately differentiated primary tumors (96.3%). No significant differences were observed in terms of age (p = 0.110), tumor site (p = 0.491), and CEA (p = 0.693) between LNM-positive and LNM-negative groups. However, significant differences were observed between LNM-positive group and LNM-negative group while considering females (p = 0.048), and patients in a more advanced T stage (p = 0.023), with poorer differentiation (p = 0.025), smaller tumor size (p = 0.024), and higher PALR (p = 0.002), and PLR (p = 0.043). **Table 1** provides detailed information regarding the different observations. When PALR was considered a continuous variable, patients in the LNM-negative group had lower PALR than those in the LNM-positive group (p = 0.006) (**Figure 2**).

**Table 1:** Clinicopathological variables of patients with cN0 colon cancer.

Variables	Total	Lymph node metastasis		P
		Positive (n = 77)	Negative (n = 192)	
Sex				0.048
Male	158	38	120	
Female	111	39	72	
Age (years)				0.110
≥ 65	129	31	98	
< 65	140	46	94	
Tumor site				0.491
Left	166	50	116	
Right	103	27	76	
T stage				0.023
T1+T2	51	8	43	
T3+T4	218	69	149	
Grade				0.025
High/Moderate	259	71	188	
Low	10	6	4	
Tumor size (cm)				0.024
Median (IQR)	4.00 (3.50-5.50)	4.00 (3.00-5.00)	4.30 (3.50-5.50)	
CEA (ng/ml)				0.693
Median (IQR)	3.13 (1.99-6.28)	3.42 (2.00-6.57)	2.97 (1.98-6.16)	
PALR				
Low	193	45	148	0.002
High	76	32	44	
PLR				
Low	48	8	40	0.043
High	221	69	152	

PLR, platelet/lymphocyte ratio; PALR, (platelet × albumin)/lymphocyte ratio; CEA, carcinoembryonic antigen; IQR, interquartile range.

### Univariate and multivariate logistic regression

**Figure 3:**
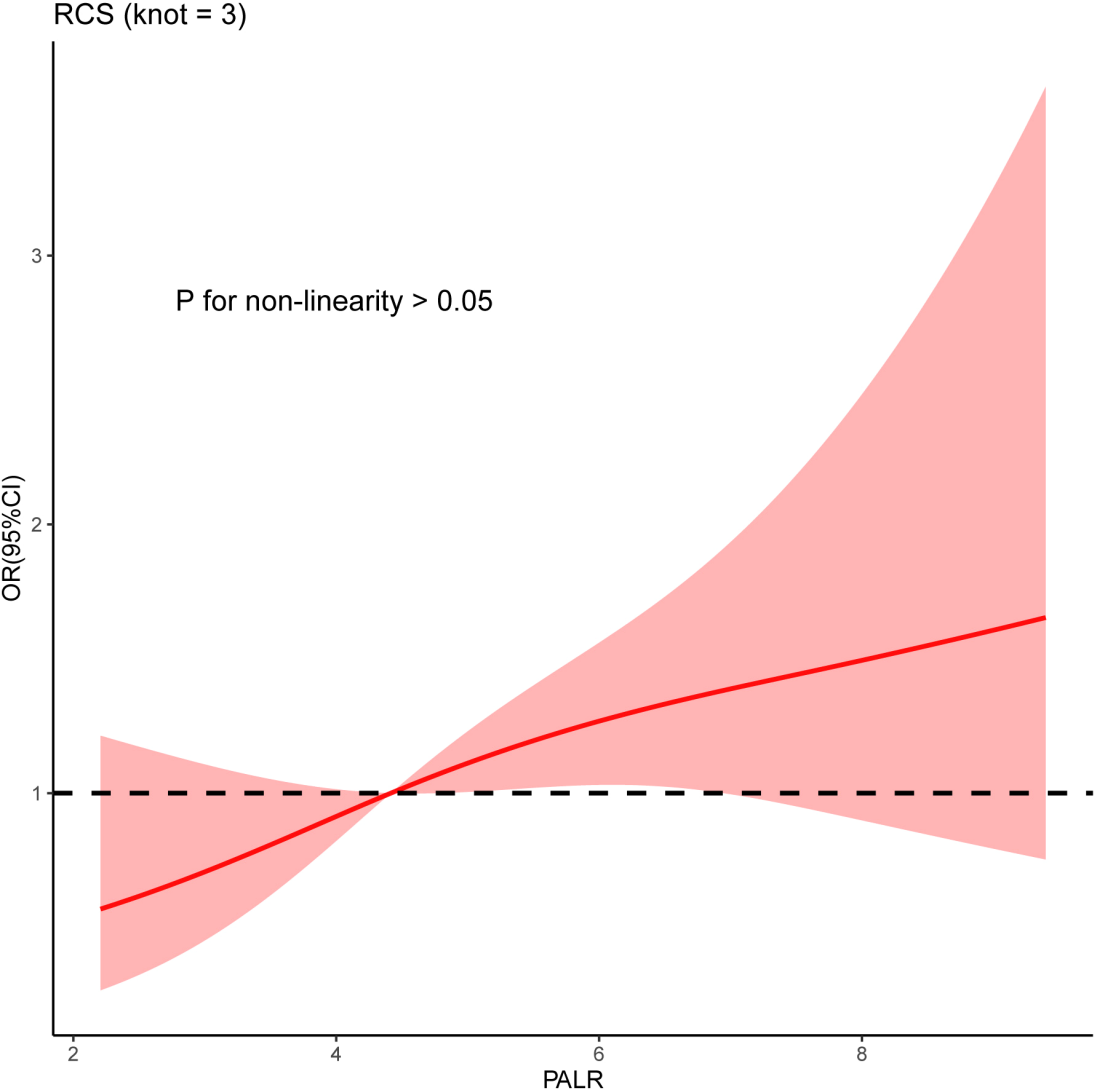
The non-linearity relationship between PALR and the risk of LNM was explored using RCS.

Univariate analysis showed that sex, T stage, grade, and PALR were correlated with LNM in patients with cN0 CC. Multivariate analysis identified sex, T stage, and PALR as the independent predictors of LNM in cN0 CC (**Table 2**). The AUC value of PALR for predicting LNM was 0.607 (95% confidence interval [CI], 0.546 - 0.666), which was significantly better than that of PLR (0.568; P < 0.001). The diagnostic sensitivity and specificity of PALR were 41.2% and 77.1%, respectively. We used RCS with three knots at the 5^th^, 50^th^, and 95^th^ centiles to develop a flexible model of the association of PALR with the risk of LNM based on multivariate analysis. In essence, the odds ratio (OR) curve exhibited an upward tendency, indicating a linear association between PALR and the risk of LNM (p for non-linearity > 0.05) and the risk of LNM increased with increasing PALR (**Figure 3**).

**Table 2:** Logistic analyses the predictors of lymph node metastasis in patients with cN0 colon cancer.

Variables	Univariate analysis		Multivariate analysis	
	OR (95% CI)	P	OR (95% CI)	P
Sex		0.049		0.048
Female	Ref		Ref	
Male	0.585 (0.342-0.997)		0.567 (0.325-0.994)	
Age (years)		0.111		
≥ 65	Ref			
< 65	0.646 (0.376-1.101)			
Tumor site		0.491		
Right	Ref			
Left	1.213 (0.704-2.122)			
T stage		0.027		0.046
T1+T2	Ref		Ref	
T3+T4	1.645 (2.489-5.966)		2.331 (1.062-5.708)	
Grade		0.037		0.072
Low	Ref		Ref	
High/Moderate	3.972 (1.103-15.925)		3.407 (0.907-14.154)	
Tumor size (cm)	0.874 (0.741-1.016)	0.092		
CEA (ng/ml)	1.004 (0.997-1.017)	0.296		
PALR		0.002		0.011
Low	Ref		Ref	
High	2.392 (1.358-4.212)		2.118 (1.182-3.786)	

CEA, carcinoembryonic antigen; PALR, (platelet × albumin)/lymphocyte ratio; OR, odds ratio; CI, confidence interval.

### Clinicopathological characteristics of cN0 CC associated with PALR

**Figure 4:**
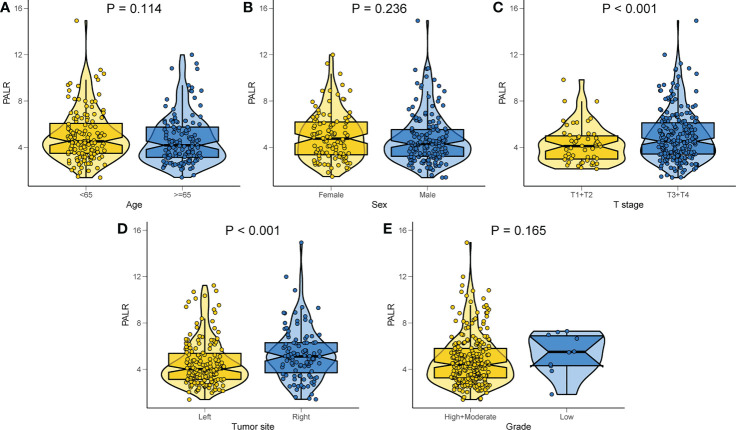
Distribution of PALR values among different clinicopathological variables. **(A)** Age, **(B)** Sex, **(C)** T stage, **(D)** Tumor site, **(E)** Grade.

Of the 269 patients, 193 were categorized as the ‘low PALR’ group while the remainder as the ‘high PALR’ group according to the optimum cut-off values. The results showed a significant association between higher PALR and the parameters including right-sided CC, larger tumor size, higher PLR, and higher LNM rate, while sex, age, T stage, grade, and CEA were not statistically correlated with higher PALR (**Table 3**). The distribution of PALR among different clinicopathological variables is shown in **Figure 4**. A higher PALR was observed in patients with right-sided CC with deeper invasion.

**Table 3:** The relationship between clinicopathological variables and PALR.

Variables	PALR		P
	Low (n = 193)	High (n = 76)	
Sex			
Male	119	39	0.121
Female	74	37	
Age (years)			
≥ 65	94	35	0.695
< 65	99	41	
Tumor site			
Left	127	39	0.028
Right	66	37	
T stage			
T1+T2	42	9	0.062
T3+T4	151	67	
Grade			
High/Moderate	187	72	
Low	6	4	
Tumor size (cm)			
Median (IQR)	4.00 (3.25-5.00)	4.50 (3.50-6.00)	0.019
CEA (ng/ml)			
Median (IQR)	3.28 (2.06-6.77)	2.57 (1.86-4.94)	0.110
PLR			
Low	48	0	< 0.001
High	145	76	
LNM			
Negative	148	44	0.002
Positive	45	32	

CEA, carcinoembryonic antigen; PALR, (platelet × albumin)/lymphocyte ratio; PLR, platelet/lymphocyte ratio; IQR, interquartile range; LNM, lymph node metastasis.

### Sensitivity analyses

Higher PALR quartiles are independently correlated with the risk of LNM in cN0 CC after adjustments for sex, T stage, and grade (p for trend = 0.041). The adjusted OR for the highest PALR versus the lowest quartile was 2.328 (95% CI, 1.060 - 5.291) for LNM (**Supplementary Table 1**).

### Construction and validation of the nomogram

**Figure 5:**
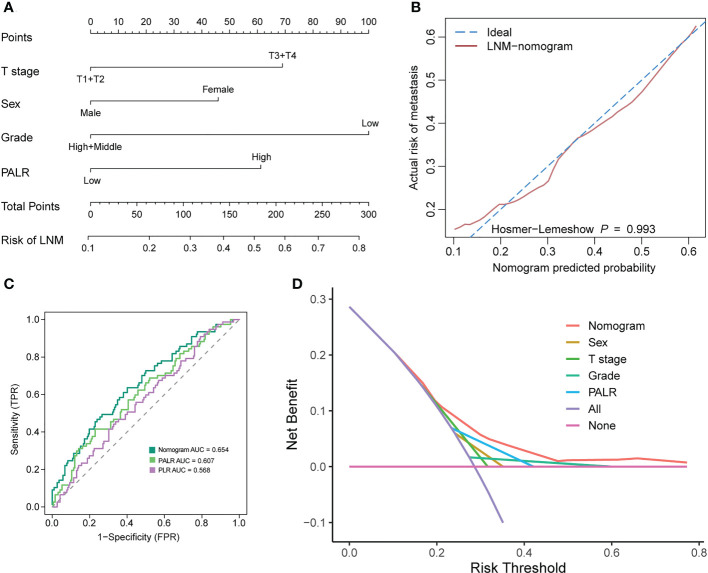
Developing and evaluating a nomogram for predicting LNM in patients with cN0 colon cancer. **(A)** nomogram, **(B)** calibration curves, **(C)** ROC curves, **(D)** Decision curve analysis.

To facilitate clinicians to calculate the risk of LNM in the individual patient with cN0 CC, we constructed a nomogram incorporating PALR. After taking into account previous research results and the biology of CC (27, 28), tumor grade was still included in our prediction model, although it was not an independent factor of LNM in our study. The indicators, including sex, T stage, grade, and PALR were selected in this model, as shown in **Figure 5A**. The adjusted C-index of nomogram after 1000 times of bootstrap resampling was 0.637. The calibration plots showed that the calibration prediction curve fits well with the ideal curve (Hosmer-Lemeshow test: p = 0.993) (**Figure 5B**). The ROC and DCA curves showed that the nomogram had a higher predictive value and net benefit compared with PALR only, indicating that this model could benefit patients in predicting the risk of LNM (**Figures 5C, D**).

## Discussion

In this study, we reported a novel preoperative biomarker, PALR, an independent predictor of LNM in patients with cN0 CC, which had a linear association with the risk of LNM. In addition, a nomogram based on PALR and clinicopathological parameters was constructed, which exhibited good performance in predicting the individual risk of LNM.

Previous studies have clarified that the systemic inflammatory response and nutritional status play important roles in tumor development and progression (29, 30). High levels of platelets are capable of promoting tumor progression and metastasis by increasing angiogenesis through the production of the vascular endothelial growth factor (31). Further, the platelet-derived growth factors have been suggested to be lymphangiogenic factors, which may either alone or jointly promote lymphatic metastasis (32). A recent study reported by Kundaktepe et al. showed a positive association of platelet counts with LNM in patients with CC (33). On the other hand, lymphocytes play a pivotal part in antitumor response by inducing cytotoxic cell death and inhibiting tumor cell proliferation and migration (34, 35). Several studies have consistently shown that tumor-infiltrating lymphocytes can kill CC cells *via* the Fas/FasL pathway (36, 37). Moreover, high platelet and low lymphocyte counts are associated with tumor progression and high PLR levels may contribute to unfavorable anti-tumor function (38).

ALB is the most abundant serum protein that reflects the nutritional status and inflammatory responses (39). Jiang et al. reported preoperative hypoalbuminemia as a risk factor for a high proportion of LNM in patients with CC (40). Inflammation or nutritional index as independent predictors of LNM have been reported in many types of tumors. In the previous studies of CC and medullary thyroid carcinoma, PLR was independently correlated with LNM (14, 41). ALB was also confirmed as a predictive biomarker of LNM in patients with gastric neuroendocrine tumor (42). Chen et al. identified that the prognostic nutritional index had an independent correlation with LNM of patients with non-small cell lung cancer (43). Our focus on the role of inflammation and nutrition in tumor progression prompted us to evaluate PALR as a novel biomarker for predicting LNM in patients with cN0 CC, which has the potential to reflect the balance between systemic inflammation and nutritional status.

The current study has several limitations. First, this study is a single-center retrospective study, and selectivity bias is inevitable. Second, due to the relatively small sample size, the persuasiveness of our findings will be compromised to some extent. Therefore, future validation studies having a large-sample size with adequate representative groups from other centers are necessary to promote the clinical application of PALR. Third, the indicators of grade and the T stage were obtained from the postoperative pathological analysis. Although the T stage and grade can be obtained preoperatively through imaging techniques and puncture, respectively, there is a possibility that the preoperative diagnosis may differ from the postoperative pathological findings (44, 45). Fourth, further follow-up of patients is warranted to investigate the relationship between PALR and overall survival.

## Conclusion

PALR is a novel promising inflammation-nutrition biomarker to predict the LNM in patients with cN0 CC. Large-scale prospective studies are required to validate our results in the future.

## Data availability statement

The raw data supporting the conclusions of this article will be made available by the authors, without undue reservation.

## Ethics statement

The studies involving human participants were reviewed and approved by Institutional Review Board of Yijishan Hospital of Wannan Medical College. Written informed consent for participation was not required for this study in accordance with the national legislation and the institutional requirements.

## Author contributions

WW designed the research. WD, CH and WW performed the study and analyzed the data. WD and WW wrote the paper and interpreted the data. WW help to revised manuscript. All authors contributed to the article and approved the submitted version.

## Supplementary material

Supplementary Table 1 Multivariate logistic analysis the predictors of LNM according to the quartile of PALR in patients with cN0 colon cancer.
