# Supplementary material for: A novel potential inflammation-nutrition biomarker for predicting lymph node metastasis in clinically node-negative colon cancer
Source: Front Oncol. 2023 Apr 4;13:995637. doi: 10.3389/fonc.2023.995637 (PMC10111825; doi:10.3389/fonc.2023.995637)
Supplement: Supplementary Table 1 — Multivariate logistic analysis the predictors of LNM according to the quartile of PALR in patients with cN0 colon cancer. [file Table_1.docx]

Supplementary Table 1. Multivariate logistic analysis the predictors of LNM according to the quartile of PALR in patients with cN0 colon cancer.

| Variables | Multivariate analysis |  |
| --- | --- | --- |
|  | OR (95% CI) | P |
| Sex |  | 0.046 |
| Female | Ref |  |
| Male | 0.567 (0.324-0.989) |  |
| T stage |  | 0.043 |
| T1+T2 | Ref |  |
| T3+T4 | 2.360 (1.076-5.772) |  |
| Grade |  | 0.082 |
| Low | Ref |  |
| High/Moderate | 3.234 (0.907-14.154) |  |
| PALR |  |  |
| 1st Quartile | Ref |  |
| 2nd Quartile | 1.409 (0.617-3.283) | 0.419 |
| 3rd Quartile | 1.491 (0.659-3.451) | 0.341 |
| 4th Quartile | 2.328 (1.060-5.291) | 0.038 |
| PALR P for trend | | 0.041 |

Multivariable-adjusted ORs and 95% CIs for LNM by the quartile of PALR with first quartile (lowest quartile) as reference. Abbreviations: PALR, (platelet × albumin) /lymphocyte ratio; OR, odds ratio; CI, confidence interval; LNM, lymph node metastasis.
